# Breast Angiosarcoma Metastatic to the Ovary

**DOI:** 10.1155/2009/381015

**Published:** 2009-06-24

**Authors:** Frederico F. Souza, Amol Katkar, Annick D. Van den Abbeele, Pamela J. Dipiro

**Affiliations:** Department of Radiology, Dana-Farber Cancer Institute, Harvard Medical school, 44 Binney Street, Boston, MA 02215, USA

## Abstract

Ovarian masses are common findings in general gynecological practice. Approximately 5%–10% of ovarian malignancies are diagnosed as metastatic tumors. Primary angiosarcoma can arise anywhere in the body and when it arises in the breast, it usually affects women in their 3rd and 4th decades and accounts for one in 1700–2300 cases of primary breast cancer. Although unusual, breast angiosarcomas tend to metastasize hematogenously rather than lymphogenously, have high rates of local recurrence, that often develop metastases soon after treatment, and have a dismal prognosis. We present a case of a solitary ovarian metastasis from angiosarcoma of the breast.

## 1. Case

The patient is a 41-year-old woman, who noticed a right breast mass in December 2004. A mammogram and ultrasound at that time were suggestive of a cyst. Repeat mammogram and ultrasound in October 2005 showed the lesion to have grown. The patient underwent needle localization and excisional biopsy, revealing a 4.9 cm intermediate grade angiosarcoma. The patient subsequently underwent chemotherapy with a total of 6 cycles of liposomal doxorubicin.

**Figure 1:**
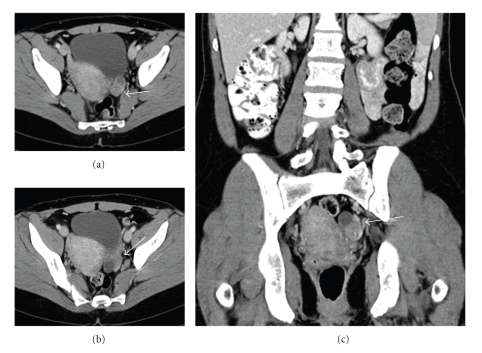
CT scan of the abdomen and pelvis (10/13/2007). Axial (a) and (b) and Coronal (c) images obtained through the pelvis demonstrate a complex solid and cystic mass in the left adnexa (whie arrow) measuring 3.2 × 2.4 cm.

**Figure 2:**
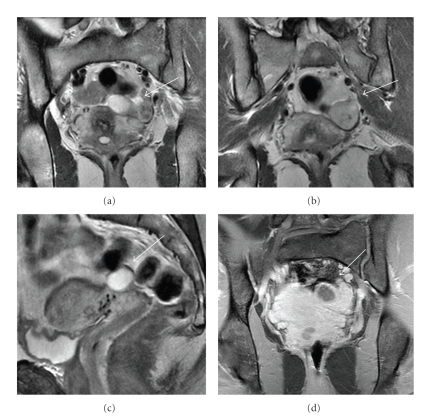
MRI of the pelvis (10/29/2007). Coronal T2-weighted images (a) and (b) demonstrate a left adnexal mixed solid and cystic mass (white arrow). The hyperintense T2 area represents the cystic component, and the isointense T2 area is the soft tissue component of the mass. Sagittal T2-weighted image (c) demonstrates the cystic component of the mass (white arrow). On coronal T1 Fat-Sat postcontrast image (d), the solid component of the mass enhances homogeneously after gadolinium administration; however, the cystic component is hypointense (white arrow.)

**Figure 3:**
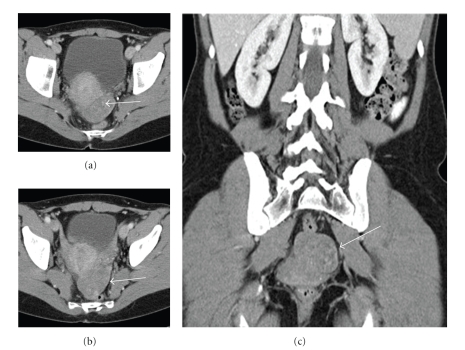
CT scan of the abdomen and pelvis (1/12/208). Axial (a) and (b) and coronal (c) images of the pelvis demonstrate a large, predominantly hypodense, left adnexal soft tissue mass (white arrow). Note that the cystic component of the mass has resolved and the soft tissue portion has significantly increased.

Restaging CT scans of the chest, abdomen, and pelvis (Figure 1), revealed a mixed cystic and solid structure in the left adnexal region, measuring 3.2 × 2.4 cm, which was felt to represent ovary with a complex cyst. Further evaluation with MRI performed 16 days later (Figure 2) revealed a lesion with high-signal intensity on T2-weighted images and low signal intensity on T1-weighted images. Repeat CT scan performed in 2 weeks (Figure 3) revealed further increase in the size of the left adnexal mass with interval resolution of the cystic component. The patient underwent laparoscopic left oophorectomy, which revealed a high-grade metastatic angiosarcoma, measuring approximately 5.2 cm and involving ovarian parenchyma and adnexal soft tissue.

## 2. Discussion

Ovaries are common sites for metastatic disease; however, metastases to the ovaries account for only 10% of ovarian cancers. Colon cancer is the most frequent primary malignancy of nongenital origin giving rise to ovarian metastases [2]. Other common primary sites that metastasize to the ovary include the stomach, breast, lung, and pancreas. The term “Krukenberg tumor” is sometimes used to refer to any metastases to the ovary. However, this term actually refers specifically to an ovarian metastasis consisting of mucin-filled signet ring cells in a cellular stroma, usually from a carcinoma of the gastric antrum [6]. 

The differential diagnosis for adnexal masses seen in patients with primary nonovarian malignancy consists of metastases, primary ovarian malignancy, or incidental benign pathology. Metastases to the ovary are typically bilateral, solid, and strongly enhance after contrast administration. Cystic and necrotic areas are common and tumors may be predominantly cystic, thus resembling primary ovarian cancer. The overlap of radiologic appearances between primary ovarian cancer and metastases to the ovaries is substantial, and imaging distinction between the two may be impossible [7]. However, Brown et al. in their series suggested that most metastatic neoplasms are predominantly solid or a mixture of cystic and solid components, in comparison with primary epithelial ovarian neoplasms, which are more likely to be predominantly cystic [8].

Megibow et al. [9] attempted to correlate the appearance of ovarian metastases with histology of the primary tumor based on the CT appearance. They reported that cystic, fluid-filled or mixed fluid and soft tissue lesions were more commonly seen in metastases from the colon. Solid metastases were seen in four of five metastases from the stomach. Necrosis in a solid ovarian lesion is suggestive of malignancy, though necrosis was not detected in their cases.

There is relatively little published information concerning the spread and frequency of metastatic sarcoma to the ovary. In a series from 1990, Young and Scully described 21 cases of metastatic sarcoma to the ovaries [10]. In their analysis, the most common primary site was the uterus (11 of 21 cases). The most common extragenital primary tumor was leiomyosarcoma of the small intestine, followed by one case each of leiomyosarcoma of the stomach, retrovesical leiomyosarcoma, fibrosarcoma of the anterior abdominal wall, sarcoma of the smooth muscle, presumed cardiac hemangiosarcoma, osteosarcoma of the maxilla, chondrosarcoma of the rib, and Ewing's sarcoma of the pubic bone.

To our knowledge, there are no case reports in the literature describing a solitary ovarian metastasis of breast angiosarcoma shown on CT and MRI. The MRI of the pelvis performed in our patient demonstrated a predominantly solid, hyperintense T2, and hypointense T1 lesion that enhanced after gadolinium administration. A peripheral cystic component in the lesion was better appreciated on the enhanced MRI images than on CT. Gadolinium-enhanced MRI is reportedly slightly superior to both contrast-enhanced CT and Doppler sonography in the characterization of adnexal masses [11]. The administration of gadolinium is important because it facilitates the differentiation between cystic and solid elements not appreciated on the precontrast T1- and T2-weighted images. 

On imaging studies, ovarian metastases may frequently resemble a primary ovarian neoplasm. Therefore, a definitive diagnosis can only be made after surgical removal of the mass and histopathologic examination.
